# Comparison of Conventional TNM and Novel TNMB Staging Systems for Non–Small Cell Lung Cancer

**DOI:** 10.1001/jamanetworkopen.2019.17062

**Published:** 2019-12-06

**Authors:** Greg J. Haro, Bonnie Sheu, Nancy R. Cook, Gavitt A. Woodard, Michael J. Mann, Johannes R. Kratz

**Affiliations:** 1 Division of Adult Cardiothoracic Surgery, University of California, San Francisco; 2 Division of Preventive Medicine, Brigham and Women’s Hospital, Harvard Medical School, Boston, Massachusetts

## Abstract

**Importance:**

Improved staging for non–small cell lung cancer (NSCLC) represents a critical unmet need. External validations of the eighth edition of the TNM staging system have yielded disappointing results, with persistently high mortality observed in early-stage disease.

**Objective:**

To determine whether incorporation of a molecular prognostic classifier into conventional TNM staging for NSCLC improves estimation of disease-free survival.

**Design, Setting, and Participants:**

This cohort study was conducted at an academic, quaternary care medical center from 2012 to 2018. A consecutive series of 238 patients underwent surgical resection of stage I to IIIC nonsquamous NSCLC and had molecular prognostic classifier testing performed. Data analysis was conducted in May 2019.

**Exposures:**

Patients were restaged according to the seventh and eighth editions of the TNM staging system and the novel TMMB staging system, which maintains the order and structure of the eighth edition of the TNM but downstages or upstages according to low or high molecular risk, respectively.

**Main Outcomes and Measures:**

The primary outcome was disease-free survival 3 years from the time of surgical resection. Reclassification statistics were then used to evaluate performance and improvement measures of the TNM seventh and eighth editions and the TNMB staging system.

**Results:**

Two hundred thirty-eight patients (144 [60.5%] female; median [interquartile range] age, 70 [63-75] years) were analyzed. The median (interquartile range) follow-up was 25 (14-40) months, and the disease-free survival rate was estimated to be 58.3% (95% CI, 45.7% to 69.0%). One hundred fifty-nine patients (66.8%) were reclassified by the TNMB staging system. Overall model fit remained the same for the seventh and eighth editions of the TNM staging system, whereas the *R*^2^ statistic (change from 0.22 to 0.31), concordance index (change from 0.68 to 0.73), and log-rank χ^2^ (change from 38 to 108) were all associated with improvements after TNMB adoption. The TNMB system, compared with the TNM eighth edition, was associated with enhanced identification of high-risk patients and better differentiation of those without recurrence from those who had recurrence (net reclassification improvement, 0.28; 95% CI, 0.08 to 0.46; *P* < .001), whereas the eighth edition compared with the seventh edition was not associated with improvement of this measure (net reclassification improvement, 0.02; 95% CI, –0.18 to 0.21; *P* = .87).

**Conclusions and Relevance:**

The TNMB staging system was associated with improved estimation of disease-free survival compared with conventional TNM staging. Incorporation of a molecular prognostic classifier into staging for NSCLC may lead to better identification of high-risk patients.

## Key Points

**Question:**

Compared with conventional TNM staging for non–small cell lung cancer, does incorporation of a molecular prognostic classifier by the TNMB staging system improve estimation of disease-free survival?

**Findings:**

In this cohort study of 238 patients who underwent surgical resection of non–small cell lung cancer, 66.8% of patients were reclassified using the molecular prognostic classifier within the TNMB staging system. The eighth edition of the TNM system, compared with the seventh edition of the TNM system, was not associated with improvement of any reclassification measures; however, compared with the TNM eighth edition, use of the TNMB system was associated with improved overall model fit, enhanced identification of high-risk patients, and better differentiation of patients without vs patients with recurrence.

**Meaning:**

The TNMB staging system may be associated with improved estimation of disease-free survival compared with conventional TNM staging.

## Teaser

This cohort study examines whether incorporation of a molecular prognostic classifier into conventional TNM staging for non–small cell lung cancer improves estimation of disease-free survival.

## Introduction

Improved staging for non–small cell lung cancer (NSCLC) represents a critical unmet need. The eighth edition of the TNM staging system (hereafter referred to as “eighth edition”) was recently adopted in 2018.^1^ Despite the advances of the molecular genetic era, the TNM system remains dependent on tumor size (T), nodal status (N), and the presence of metastasis (M). Importantly, external validations of the eighth edition have failed to show improvement in estimation of survival. In particular, risk stratification of early-stage disease continues to underperform, with 5-year overall mortality rates as high as 40% to 45% in patients with pathologic stage I disease.^2,3^

Because of the heterogeneity in outcomes for early-stage disease, our group and others have previously suggested that further refinement to staging may not be possible without adoption of biological predictors.^4^ We previously reported the largest and most comprehensive independent validation of a molecular prognostic classifier of tumor biology in NSCLC.^5,6,7^ The molecular prognostic classifier reliably identifies patients with early-stage, nonsquamous NSCLC at high risk for mortality after surgical resection.

On the basis of an international, multicenter cohort of 1373 patients from 1997 to 2007, we then developed and validated a novel staging system, TNMB (with “B” denoting “biology”), that incorporated the molecular prognostic classifier into the eighth edition.^8^ The TNMB staging system maintains the order and structure of the eighth edition, but downstages or upstages patients if they are found to be at low or high molecular risk, respectively (Table 1). In that retrospective analysis,^8^ survival estimations remained unchanged after adoption of each successive conventional staging system from the sixth to eighth editions of the TNM. In contrast, the addition of the molecular prognostic classifier within the TNMB staging system was associated with improved overall estimation by all reclassification metrics.

**Table 1.** TNMB Staging System^a^

TNM Eighth Edition Stage and Molecular Prognostic Classifier	TNMB Stage
Stage IA1	
Low-risk	Stage IA1
Intermediate-risk	Stage IA2
High-risk	Stage IA3
Stage IA2	
Low-risk	Stage IA1
Intermediate-risk	Stage IA2
High-risk	Stage IA3
Stage IA3	
Low-risk	Stage IA2
Intermediate-risk	Stage IA3
High-risk	Stage IB
Stage IB	
Low-risk	Stage IA3
Intermediate-risk	Stage IB
High-risk	Stage IIA
Stage IIA	
Low-risk	Stage IB
Intermediate-risk	Stage IIA
High-risk	Stage IIB
Stage IIB	
Low-risk	Stage IIA
Intermediate-risk	Stage IIB
High-risk	Stage IIIA
Stage IIIA	
Low-risk	Stage IIB
Intermediate-risk	Stage IIIA
High-risk	Stage IIIB
Stage IIIB	
Low-risk	Stage IIIA
Intermediate-risk	Stage IIIB
High-risk	Stage IIIC
Stage IIIC	
Low-risk	Stage IIIB
Intermediate-risk	Stage IIIC
High-risk	Stage IIIC

^a^ The TNMB staging system (with “B” denoting “biology”) maintains the order and priority of the eighth edition of the TNM staging system for non–small cell lung cancer but allows for modifications according to the molecular prognostic classifier, which provides an estimate of low, intermediate, and high risk of mortality. Patients are upstaged by 1 stage for high risk, downstaged by 1 stage for low risk, and there is no change in stage for intermediate risk.

In this study, we present a prospective validation of the TNMB staging system in an independent cohort of 238 patients who underwent surgical resection of stage I to IIIC, nonsquamous NSCLC with molecular prognostic classifier testing at the University of California, San Francisco from 2012 to 2018. Patients were restaged according to the seventh edition of the TNM (hereafter referred to as “seventh edition”), the eighth edition, and the TMMB staging system, and reclassification statistics were then used to evaluate performance and improvement measures of each of the staging systems.

## Methods

### Prospective Patient Cohort

This cohort study prospectively followed 238 patients who underwent surgical resection of stage I to IIIC nonsquamous NSCLC with molecular prognostic classifier testing during the time of cancer treatment at University of California, San Francisco from 2012 to 2018. All patients were treated with curative intent per National Comprehensive Cancer Network guidelines.^9^ Patients were restaged using pathologic tumor size and nodal status according to the seventh edition, the eighth edition, and the TMMB staging system.^8^

The primary outcome was disease-free survival 3 years from the time of surgical resection. Recurrence and vital status were prospectively obtained from patient contact, medical records, and the cancer center’s database. Reclassification statistics were used to evaluate performance and improvement measures of each of the staging systems.

Patients found to have adenocarcinoma in situ or who developed second primary NSCLC were excluded from analysis. Patients who received neoadjuvant or adjuvant therapy according to National Comprehensive Cancer Network guidelines were included in this study, but those who received adjuvant therapy solely on the basis of the molecular prognostic classifier were excluded because adjuvant therapy has been shown to improve the prognosis of high-risk patients.^10,11^

The study was approved by the University of California, San Francisco institutional review board, and written informed consent was obtained from all study participants. Race was self-classified by study participants and is presented in this study because there are well-known differences in disease heterogeneity among races. This prospective cohort study follows the Strengthening the Reporting of Observational Studies in Epidemiology (STROBE) reporting guideline.

### TNMB Staging System

 The TNMB system incorporates a molecular prognostic classifier into the eighth edition.^5,6,7,8^ The molecular prognostic classifier was developed in our laboratory and integrates expression levels of 11 cancer-related target genes (*BAG1*,* BRCA1*,* CDC6*,* CDK2AP1*,* ERBB3*,* FUT3*,* IL11*,* LCK*,* RND3*,* SH3BGR*, and* WNT3A*) against a background of 3 reference genes (*ESD*,* TBP*, and* YAP1*). The molecular prognostic classifier provides an estimate of low, intermediate, and high risk of mortality.

### Statistical Analysis

Disease-free survival was defined as the time from surgical resection to recurrence, death, or censoring. Patients were administratively censored 3 years after surgical resection, and it was assumed that censoring was independent of survival time. The Kaplan-Meier estimate and log-rank test were used to evaluate disease-free survival in Stata statistical software version 15.1 (StataCorp). Overall model performance was assessed by the concordance index (C-index) and the Royston modification of the Nagelkerke *R*^2^ statistic using stcoxgrp and str2ph, respectively, in Stata version 15.1.^12^

The categorical net reclassification improvement (NRI) was calculated from the survival probabilities of the ordered staging systems among those moving up and down the stage classifications.^13^ The NRI was calculated using survIDINRI in R statistical software version 3.4.4 (R Project for Statistical Computing).^14^

For all statistical tests, a prespecified 2-sided α = .05 was considered significant. All analyses were performed in Stata statistical software version 15.1 unless otherwise noted. Of note, adoption of the eighth edition refers to a comparison of the eighth vs seventh editions, and adoption of TNMB refers to a comparison of TNMB vs the eighth edition. Data analysis was conducted in May 2019.

## Results

Two hundred thirty-eight patients underwent surgical resection of stage I to IIIC nonsquamous NSCLC with curative intent and underwent molecular prognostic classifier testing (Table 2). The median (interquartile range) age was 70 (63-75) years, and patients were predominantly female (144 [60.5%]), white (159 [66.8%]), and had a history of smoking (159 [66.8%]). The main histologic subtype was adenocarcinoma (231 [97.1%]), and more than 75% had stage I disease. Adjunctive treatment was given to all patients per National Comprehensive Cancer Network recommendations. Twenty-two patients (9.5%) received neoadjuvant therapy, and 50 patients (21.0%) received adjuvant therapy. The cohort had a median (interquartile range) follow-up of 25 (14-40) months, and 3-year disease-free survival rate was estimated to be 58.3% (95% CI, 45.7%-69.0%).

**Table 2.** Summary of Patient and Tumor Characteristics

Characteristic	Patients, No. (%)
No.	238
Age, median (IQR), y	70 (63-75)
Female	144 (60.5)
Race	
White	159 (66.8)
Asian	52 (21.9)
Other	27 (11.3)
Smoking history	159 (66.8)
Lung cancer subtype	
Adenocarcinoma	231 (97.1)
Large cell carcinoma	3 (1.3)
Not otherwise specified	4 (1.7)
Therapy	
Neoadjuvant	22 (9.5)
Adjuvant	50 (21.0)
Follow-up, median (IQR), mo	25 (14-40)
3-y disease-free survival, % (95% CI)	58.3 (45.7-69.0)

Abbreviation: IQR, interquartile range.

Because of the time frame of the study, patients from 2012 to 2017 were formally staged according to the seventh edition, and patients from 2018 were formally staged according to the eighth edition after its adoption. All patients were then restaged according to the seventh edition, eighth edition, and TNMB staging systems to allow for comparison of survival estimation (Table 3). On the basis of the molecular prognostic classifier, 124 (52.1%), 67 (28.1%), and 47 (20.0%) patients were found to have low, intermediate, and high risk of mortality, respectively. One hundred fifty-nine patients (66.8%) were reclassified by the TNMB staging system. Incorporation of the molecular prognostic classifier within the TNMB staging system led to both upward and downward classification of patients.

**Table 3.** Staging Reclassification by the Seventh and Eighth Editions of the TNM Staging System and the TNMB Staging System for Non–Small Cell Lung Cancer^a^

Overall Stage	Patients, No. (%)		
	TNM Seventh Edition	TNM Eighth Edition	TNMB
Stage I			
IA1	154 (64.7)	18 (7.6)	71 (29.8)
IA2	NA	89 (37.4)	56 (23.5)
IA3	NA	47 (20.0)	30 (12.6)
IB	49 (20.6)	33 (13.9)	25 (10.5)
Stage II			
IIA	15 (6.3)	16 (6.7)	21 (8.8)
IIB	6 (2.5)	16 (6.7)	16 (6.7)
Stage III			
IIIA	14 (5.9)	18 (7.6)	7 (2.9)
IIIB	0	1 (0.4)	11 (4.6)
IIIC	NA	0	1 (0.4)

Abbreviation: NA, not applicable.

^a^ The TNMB (with “B” denoting “biology”) is a novel staging system that integrates a validated 14-gene molecular prognostic classifier into the eighth edition of the TNM staging System. The table depicts the reclassification of patients from the TNM seventh edition into the TNM eighth edition and TNMB staging systems.

The TNMB staging system was associated with improved overall model fit compared with conventional staging (Table 4). As opposed to a minimal change in the modified Nagelkerke *R*^2^ statistic with adoption of the eighth edition (seventh edition *R*^2^ = 0.21; eighth edition *R*^2^ = 0.22), TNMB staging was associated with superior model fit demonstrated by an increase of the *R*^2^ to 0.31. Similarly, the log-rank χ^2^ statistic for trend by stage decreased after adoption of the eighth edition (seventh edition χ^2^ = 85; eighth edition χ^2^ = 38), but increased after adoption of the TNMB staging system (χ^2^ = 108). Moreover, the C-index, or area under the receiver operating characteristic curve, remained static after adoption of the eighth edition (seventh edition C-index = 0.68; eighth edition C-index = 0.68), but increased to 0.73 in TNMB staging. These findings illustrate that TNMB is associated with an improved overall model fit as well as improved discrimination compared with conventional staging.

**Table 4.** Performance and Improvement Measures From Adoption of the Seventh and Eighth Editions of the TNM Staging System and the TNMB Staging System for Non–Small Cell Lung Cancer^a^

Performance Measures	TNM Seventh Edition	TNM Eighth Edition	TNMB
Nagelkerke *R*^2^^b^	0.21	0.22	0.31
C-index	0.68	0.68	0.73
Log-rank χ^2^	85	38	108
NRI (95% CI)^c^	Eighth edition vs seventh edition, 0.02 (–0.18 to 0.21)^d^	TNMB vs eighth edition, 0.28 (0.08 to 0.46)^e^	
Event	0.54	0.73	
Nonevent	0.52	0.45	

Abbreviations: C-index, concordance index (area under the receiver operating characteristic curve); NRI, net reclassification improvement.

^a^ The TNMB (with “B” denoting “biology”) is a novel staging system that integrates a validated 14-gene molecular prognostic classifier into the eighth edition of the TNM staging system. The TNMB system had improved model fit and reclassification compared with the seventh and eighth editions of the TNM. There were no substantial changes in any of these measures after adoption of conventional staging systems.

^b^ Royston modification of Nagelkerke *R*^2^.

^c^ NRI = [*P*(event|up) − *P*(event)] · *P*(up) + [*P*(event) − *P*(event|down)] · *P*(down) / *P*(event) · [1 − *P*(event)].

^d^ *P* = .87.

^e^ *P* < .001.

The NRI was also assessed from adoption of each of the staging systems. Compared with the seventh edition, staging with the eighth edition was not associated with a change in the NRI (NRI, 0.02; 95% CI –0.18 to 0.21; *P* = .87). In contrast, adoption of TNMB staging compared with the eighth edition showed a significant increase in the NRI to 0.28 (95% CI, 0.08 to 0.46; *P* < .001). This finding demonstrates that TNMB staging was associated with improved identification of high-risk patients and differentiation of those with recurrence from those without recurrence.

## Discussion

Heterogeneity in outcomes for early-stage disease in NSCLC persists despite ongoing revisions to traditional TNM staging. Many patients who were thought to have early-stage disease never received adjuvant therapy but later proved to have metastasis. Although the recently adopted eighth edition staging system was developed on the largest and most comprehensive data set to date, external validations have failed to show improvement in survival estimation.^2,3^ This suggests that further refinement to staging may not be possible with continued use of traditional clinicopathologic characteristics.

We previously developed and validated a novel staging system, TNMB, from a retrospective cohort that incorporated a robust molecular prognostic classifier of tumor biology into the eighth edition.^8^ The TNMB staging system maintains the order and priority of conventional staging, but downstages or upstages patients found to be at low or high molecular risk, respectively. The TNMB staging system was used because it is simple, transparent, and clinically intuitive. The incorporation of a fourth category representing biology is easy for clinicians to apply, and it allows for flexibility to test and incorporate future refinements from novel characterizations of tumor biology.

In this study, the TNMB staging system was prospectively validated on an independent cohort of 238 patients who underwent surgical resection of NSCLC with curative intent at University of California, San Francisco from 2012 to 2018. Incorporation of a molecular prognostic classifier by the TNMB staging system was associated with improved survival estimation compared with conventional staging. Overall model fit remained the same after adoption of recent revisions to conventional staging, whereas the *R*^2^ statistic, C-index, and log-rank χ^2^ all improved after TNMB adoption. Moreover, the TNMB system was associated with enhanced NRI, whereas there was no change in this measure following adoption of the eighth edition. Importantly, the TNMB system was associated with improved identification of high-risk patients and was better able to differentiate between patients with or without recurrence compared with conventional staging.

### Limitations

This study has several limitations. First, because of the sample size of patients in this study, we were not able to provide data regarding the agreement in observed and estimated risks of recurrence or death between staging systems, nor were we able to assess progression-free survival by Kaplan-Meier analysis because of the large number of risk categories in the eighth edition and TNMB staging systems. Second, because ours is a quaternary care center, follow-up data from patients subsequently receiving care locally could have been inaccurate despite our use of multiple redundant methods to obtain follow-up data. Third, some of our patients who were staged using the seventh edition at the time of surgical resection did not have information regarding the size of the lepidic component or invasion of surrounding structures. Because this information is used in the eighth edition, a small number of patients may, therefore, have been staged inaccurately.

## Conclusions

TNMB staging was associated with improved risk discrimination in a prospective cohort of patients with lung cancer. The addition of information from a molecular prognostic classifier resulted in improved model fit and better estimations of disease-free survival compared with conventional TNM staging. The molecular classifier and TNMB staging system used in this study may serve as the basis for the modification of subsequent staging systems for NSCLC and may enable more effective application of adjuvant therapy in patients with early-stage disease.
